# Splice choices at the threshold of activation: Alternative splicing fine-tunes Notch signaling in muscle stem cells

**DOI:** 10.1016/j.stemcr.2025.102782

**Published:** 2026-01-13

**Authors:** Holly Jiogo, Colin Crist

**Affiliations:** 1 Department of Human Genetics, McGill University, 3640 University St., Montréal H3A 0C7, Canada; 2 Lady Davis Institute for Medical Research, Jewish General Hospital, 3755 chemin de la Cote Ste. Catherine, Montréal H3T 1E2, Canada

## Abstract

Alternative splicing events have emerged as a rapid regulatory layer in gene expression. Lin et al. demonstrate that alternative splicing is widespread during muscle stem cell activation. Its functional importance is illustrated through an RNA-binding fox 1-homolog 2 (RBFOX2)-dependent splice choice in the Notch regulator Numb, showing how inclusion of a single exon can tune Notch signaling to regulate the transition from quiescence to activation.

## Main text

Muscle stem cells (MuSCs), commonly referred to as “satellite cells” for their anatomical position orbital to the myofiber and underneath the basal lamina, are responsible for postnatal growth and regeneration of skeletal muscle (Mauro, 1961). These rare cells are mitotically quiescent but “primed” to rapidly activate the myogenic program and the cell cycle to generate large numbers of myoblasts needed to fuse and regenerate the myofiber. A subset of activated MuSCs self-renew to restore the MuSC compartment, thereby fulfilling the operational definition of an adult stem cell. The transition of MuSCs from quiescence to activation is tightly regulated, combining signaling, epigenetic, transcriptional, and post-transcriptional mechanisms.

Alternative splicing of pre-mRNA is a fundamental mechanism by which eukaryotic cells mold their proteome without necessarily requiring new transcription. This mechanism imparts upon the cell a rapid ability to change cell activity in response to changes in the environment (Wright et al., 2022). Although there is emerging evidence for alternative splicing events regulating MuSC biology, these studies are restricted to individual pre-mRNAs (Chang et al., 2018) or specific regulators of splicing (Liu et al., 2020, 2025, 2020). The overall role of alternative splicing in MuSC biology remains poorly defined.

**Figure 1:**
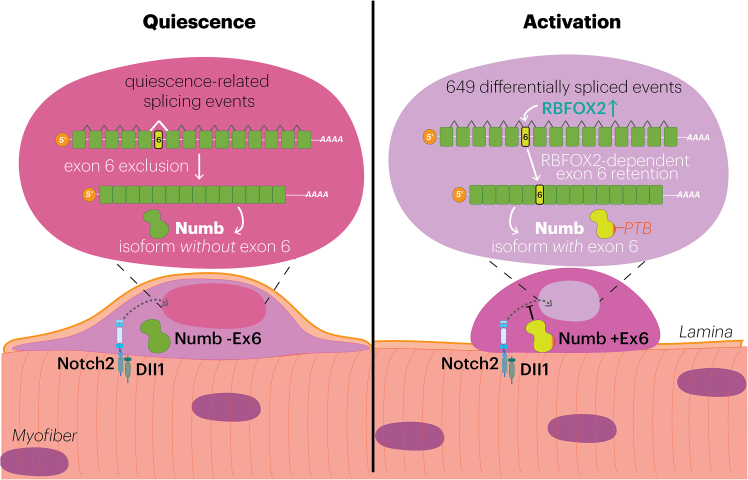
A model based on Lin et al.’s findings showing that RBFOX2 expression in activated MuSCs is associated with exon-6 inclusion in *Numb* mRNA The resulting NUMB protein isoform (Numb +Ex6) dampens Notch signaling, a pathway that would otherwise maintain MuSC quiescence.

In this issue of *Stem Cell Reports*, Lin et al. (2025) reveal how the transcriptome rapidly changes as MuSCs exit quiescence and enter the early stages of activation. They reveal that rapid and extensive splicing changes accompany MuSC transitions from a state of quiescence to the earliest steps of activation. The change in splicing is regulated in part through accumulation of RNA-binding fox 1-homolog 2 (RBFOX2), an evolutionary conserved regulator of alternative splicing (Baraniak et al., 2006). *Rbfox2* is critical for MuSCs to activate and regenerate skeletal muscle. The importance of RBFOX2 regulation of alternative splicing is illustrated by its regulation of *Numb* pre-mRNA splicing, which in turn is a key regulator of the Notch signaling pathway that regulates MuSC quiescence. RBFOX2 promotes the inclusion of *Numb* exon 6, which encodes a NUMB isoform that functions to dampen Notch signaling as a key first step to initiate MuSC activation (Figure 1).

Focusing on the transitions between quiescence, early activation, and full activation, the authors used their recently developed method of *in situ* fixation followed by cell sorting to isolate cells corresponding to quiescent, early activated, and fully activated MuSCs (Yue et al., 2020). They performed RNA sequencing (RNA-seq) on these populations to identify differentially spliced events (DSEs) using replicate multivariate analysis of transcript splicing (rMATS), a statistical analysis of RNA-seq data to identify alternatively spliced exons between experimental groups (Shen et al., 2014). 644 and 829 DSEs were identified from quiescence to early activation, and early to full activation, respectively. Most DSEs at early activation were found to be distinct from the DSEs identified at full activation, despite these cell transitions being only hours apart.

Next, the authors sought to identify a regulator of alternative splicing in MuSCs. They identified RBFOX2 protein to be significantly upregulated in activated MuSCs. They additionally show that conditional *Rbfox2* deletion in MuSCs delays their activation, with consequent delay in muscle regeneration *in vivo*.

To investigate how RBFOX2 regulates MuSC activity by alternative splicing, the authors analyzed DSEs in activated MuSCs isolated from skeletal muscle of wild-type and MuSC-specific *Rbfox2*-knockout mice. Only a small proportion of the total DSEs identified in activated MuSCs are dependent on RBFOX2, suggesting that additional regulators of alternative splicing are involved. Lin et al. (2025) identify Numb as an RBFOX2-dependent splicing target, with exon 6 undergoing rapid inclusion during MuSC activation, thereby providing a potential mechanism by which RBFOX2 influences early activation decisions. Alternative splicing of Numb generates NUMB isoforms with distinct effects on Notch signaling, proliferation, and differentiation (Dho et al., 2025).

Exon 6 encodes a portion of NUMB’s phosphotyrosine-binding domain, potentially altering how NUMB interacts with regulators of Notch receptor trafficking (Dho et al., 2025). Exon 6 inclusion would strengthen NUMB’s ability to suppress the Notch signaling needed to maintain MuSC quiescence. To test this, Lin et al. (2025) incorporated vivo-morpholinos to prevent the inclusion of exon 6. When delivered at the time of MuSC activation, these *vivo*-morpholinos effectively excluded *Numb* exon 6, resulting in perduring expression of Notch target genes, with a consequent delay in MuSC activation and re-entry into the cell cycle.

Emerging from this study is a new model by which alternative splicing acts as a rapid and widespread regulatory mechanism in stem cell activation. Within only a few hours of injury, MuSCs undergo hundreds of splicing changes, indicating that exon inclusion or exclusion can rapidly and pervasively alter the stem cell proteome. In the activation of stem cells during tissue repair, timing and energy resources matter. While transcriptional and epigenetic programs may be reinforced over many hours and days, splicing decisions can remodel the proteome almost immediately, since all the upstream regulatory events to generate a pre-mRNA have already occurred. Therefore, alternative splicing has the potential to provide a rapid mechanism to instruct how stem cells respond to tissue damage.

This concept is illustrated by RBFOX2-dependent alternative splicing of Numb, a classical and well-characterized regulator of Notch signaling. Inclusion of exon 6 alters Numb’s phosphotyrosine-binding domain to strengthen its ability to suppress Notch activity, thereby “releasing the brakes” on stem cell activation and subsequent entry into the cell cycle. Lin et al. (2025) highlight how a single exon inclusion event modifies a key regulator of stem cell activation. Given the widespread changes in exon usage accompanying MuSC activation, the study reveals how alternative splicing could be considered a core and rapid regulator of stem cell activity.

Although the work is exceptional for its contribution to fundamental knowledge, it also has potential to open some new translational avenues. For example, selectively modifying splicing factor activity via antisense oligonucleotides or small molecules could emerge to alter stem cell activation *ex vivo* and *in vivo*.

The alternative splicing mechanism revealed by Lin et al. (2025) opens new conceptual space around alternative splicing along with interesting questions that can be addressed in future studies. Only a small fraction of exon choices are RBFOX2 dependent. Are there additional splicing factors that contribute to MuSC activation? Previous studies have identified RBFOX2 as a regulator of myogenic differentiation and fusion (Singh et al., 2014). What regulates RBFOX2 activity across the myogenic program? The study also reveals that hundreds of exon choices are associated with MuSC activation but highlights Numb exon 6 inclusion as an illustrative example with biological significance. Are there additional “regulatory exons” that operate in parallel to release the brakes on MuSC activation?

In summary, Lin et al. (2025) provide an elegant study, rich in data and supported by multiple lines of evidence, establishing alternative splicing alongside epigenetic, transcriptional, and well-characterized post-transcriptional regulatory layers in stem cell biology. A single exon inclusion in *Numb* exemplifies the potential impact of alternative splicing, but the extensive alternative splicing observed during MuSC activation suggests a broader regulatory role. This study should encourage renewed attention to alternative splicing in stem cell biology.

## Declaration of interests

The authors declare no competing interests.
